# Research on Jamming Recognition Based on Time-Frequency Domain Weighted Fusion and Attention Mechanism

**DOI:** 10.3390/s25206345

**Published:** 2025-10-14

**Authors:** Yao Zhang, Shuai Wang, Lingyi Wu, Yunchao Yao, Xi Pan

**Affiliations:** School of Mechatronical Engineering, Beijing Institute of Technology, Beijing 100081, China; 3120245157@bit.edu.cn (Y.Z.); 3220235033@bit.edu.cn (S.W.); 3120225163@bit.edu.cn (L.W.); 3220240205@bit.edu.cn (Y.Y.)

**Keywords:** multi-terrain random fluctuation model, jamming recognition, time-frequency domain weighted fusion, attention mechanism

## Abstract

The jamming recognition of target detection aims to achieve rapid judgment and effective response to jamming by analyzing the target echo signals. This paper addresses the shortcomings of the existing methods in terms of jamming recognition capabilities and practical effectiveness and conducts research on jamming recognition based on time-frequency domain fusion and attention mechanism. First, by analyzing the principles of target detection and jamming effects, a multi-terrain random fluctuation model for ground detection is established. Second, the time-frequency domain weighted fusion method is proposed. Taking multi-period time domain + time-frequency domain as jamming recognition information, combined with the attention mechanism, the jamming recognition model based on time-frequency domain weighted fusion and the attention mechanism (TFWF-AM) is established. Then, the single jamming and compound jamming sample sets are established by superimposing the beating signals of target echo and multi-jamming. Finally, the accuracy of the TFWF-AM jamming recognition model is compared with that of existing method models, and the effectiveness of multi-period time domain + time-frequency domain information is verified. The results show that the TFWF-AM jamming recognition model has the highest accuracy for both single jamming and compound jamming, reaching 99.92% and 99.56%, respectively, which is 10.42% and 52.81% higher than that of the feature fusion model. This research holds huge significance for the perception and decision-making of target detection systems in complex electromagnetic environments.

## 1. Introduction

The radio detection system can obtain the target information based on the difference of radio physical field characteristics response, which has the advantages of being simple and reliable, having good range cut-off characteristics, and having low interception rate. However, it is vulnerable to human and natural jamming, which weakens or even destroys the characteristics of the target, affects the stability and reliability of the working process, and makes it impossible for the ammunition to maximize its damage ability [1]. To enhance the reliability of target detection, by identifying different types and parameters of jamming, different jamming suppression and anti-jamming strategies can be adopted to reduce signal distortion and attenuation [2].

The traditional anti-jamming method includes three aspects: selection of physical field and working principle, improvement in signal processing level, and tactical application [3]. Among them, the selection of physical field and working principle is the most effective anti-jamming method. For example, the anti-jamming ability, target resolution, and positioning accuracy are improved by selecting a narrow detection beam. The application of multiple physical fields or principles to detect targets makes it difficult for jammers to effectively interfere with the compound detectors at the same time. Increasing the complexity of the transmitted waveform makes it difficult for the enemy to analyze its characteristics. Improving signal processing level is another effective method for anti-jamming. This method uses the characteristics of target signal amplitude, increase rate, frequency, and signal duration to improve the difference between the target and jamming signals to achieve the purpose of anti-jamming. In addition, the anti-jamming ability is strengthened in tactical application through long-distance power connection and cross-use of detectors with different principles. However, the traditional theoretical calculation method of target detection is prone to failure under the condition of low signal-to-noise ratio and strong jamming [4,5]. Therefore, it is necessary to anticipate the type of jamming in advance and purposefully optimize the detection signal waveform to reduce the negative impact of jamming on target detection accuracy [6,7].

In order to enhance the adaptive perception ability of the target detection system, many experts and scholars use time-frequency domain analysis methods to extract useful information from complex signals for jamming recognition and establish a prior knowledge base based on a deep learning model. Reference [8] and Reference [9] used deep learning methods to identify a variety of single jamming and compound jamming. Gao et al. [10] proposed a target recognition algorithm based on multi-source compound imaging and deep learning to improve the recognition accuracy under smoke, dust, and camouflage jamming. Guo et al. [11] proposed a multi-domain shallow feature-guided radar active jamming multi-modal comparison recognition method, which realized accurate jamming recognition under extremely small sample conditions. Nie et al. [12] proposed an active jamming suppression method for spaceborne SAR images based on region feature refinement perceptual learning, which can effectively identify and suppress a variety of typical active blanket jamming. Guo et al. [13] proposed a lightweight ML-SNet radar compound jamming recognition algorithm with low computational complexity and good recognition performance. Gao et al. [14] proposed a jamming pattern open set recognition method based on hyperspherical 3-tuple coding, which uses a meta-recognition classifier to accurately complete the jamming pattern open set recognition task. Yang et al. [15] proposed a jamming recognition method based on multi-domain features and Transformer to achieve the purpose of jamming recognition from mixed signals. Dai et al. [16] proposed an adaptive target and jamming recognition method for Doppler radar fuze based on time-frequency joint feature and online update. In order to analyze the jamming characteristics, Ping et al. [17] proposed a modulation recognition method based on cyclic spectrum features and high-order cumulant features, which realized the robust recognition of multi-input and multi-output signals under complex channel environments. References [18,19,20] realize automatic modulation classification of signals based on convolutional neural networks. However, the existing methods only rely on point sources or simple planar targets to generate echo signals, and they also fail to consider the superposition of jamming signals and target echo signals, resulting in a significant deviation from the actual situation. Moreover, the jamming recognition model based on multi-source information fusion is a fusion of signal features but lacks research on decision-level fusion. Generally, the differences among various types of jamming are almost indistinguishable. In this paper, the differences are transformed into significant feature distances in a high-dimensional space through the attention mechanism.

Therefore, this paper addresses the problem of jamming recognition in complex electromagnetic environments and establishes a multi-terrain random fluctuation model for target detection systems, determines effective jamming recognition information, and conducts research on jamming recognition based on time-frequency domain fusion and attention mechanisms. The contributions of this paper are fourfold.

1. The multi-terrain random fluctuation model is established.
2. A multi-period “time domain + time-frequency domain” complementary jamming recognition information construction method is proposed.
3. The TFWF-AM jamming recognition model is established by integrating the attention mechanism with the time-frequency fusion method.
4. By superimposing the target echo beating signals and the multi-jamming beating signals, the single jamming and compound jamming recognition sample sets are established, respectively.

The structure of this paper is as follows: Section 1 is the introduction; Section 2 constructs the multi-terrain random fluctuation model; Section 3 establishes the jamming recognition models; Section 4 establishes the jamming recognition sample set; Section 5 compares the performance of the model; and Section 6 presents the conclusion of this paper.

## 2. Construction of Multi-Terrain Random Fluctuation Model

According to the principle of radio target detection, the jamming signals enter the receiver with the echo signals, undergo frequency mixing with the transmitting signals, pass through the low-pass filter, and extract the target and jamming information through signal processing. This section establishes a multi-terrain random fluctuation model for target detection and derives the effective beating signals of target echo. The output of the model can be directly used at the signal processing end.

### 2.1. Establishment of Multi-Terrain Detection Model

**Figure 1:**
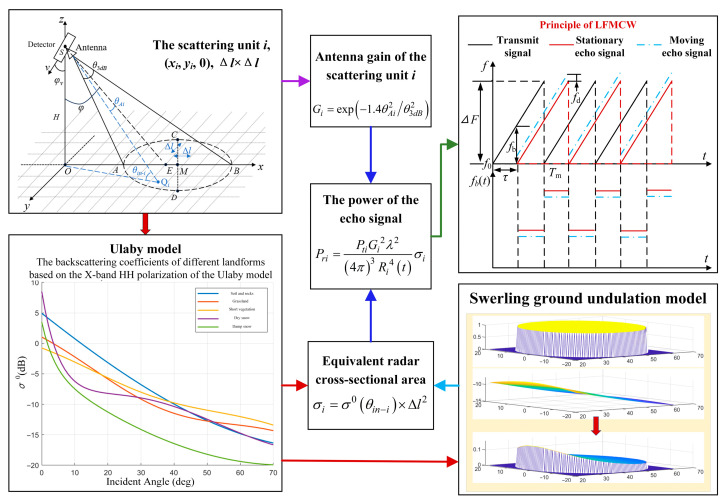
Establishment of multi-terrain echo model.

According to the principle of radio target detection, the establishment process of the ground echo model is shown in Figure 1.

First, the boundary of the ground area at a fixed height is calculated according to the relevant parameters of the detector and the antenna. The ground echo area is divided into multiple scattering units with side length Δ*l* by using the square uniform segmentation method, and the center coordinates (*x_i_*, *y_i_*, 0) of each scattering unit are calculated. Then, the radar cross-sectional area *σ*_i_ and the corresponding antenna gain *G_i_* (*θ_i_*, *φ*) of each scattering unit relative to the detector are determined by combining the Ulaby model [21] and the Swerling fluctuation model [22]. Finally, the echo power *P_ri_* of each scattering unit can be calculated.

The Ulaby backscattering characteristic model is an empirical statistical model obtained by collecting and analyzing a large amount of different terrain data. The backscattering coefficients can be obtained from Equation (1).

(1) $$\sigma^{0}\left(\theta \right)=P_{1}+P_{2}\exp\left(-P_{3}\theta \right)+P_{4}\cos\left(P_{5}\theta +P_{6}\right)$$

where *σ*^0^(*θ*) is the backscattering coefficient, measured in dB; *θ* is the local incident angle, measured in radians; and *P*_1_–*P*_6_ are the fitting parameters of the Ulaby model.

According to the Swerling ground fluctuation model, the echo region can be regarded as a large number of approximately equal scattering units in this study, so the ground random fluctuation can be regarded as an exponential distribution:

(2) $$p\left(\sigma^{0}\right)=\frac{1}{\overset{-}{\sigma^{0}}}\exp\left(-\frac{\sigma^{0}}{\overset{-}{\sigma^{0}}}\right),\sigma \;\geq \;0$$

where $\sigma^{0}$ is determined by the Ulaby model and $\overset{-}{\sigma^{0}}$ is the mean value of the fluctuation process.

For a point target, the time domain waveform of the transmitted signal for the sawtooth frequency modulation can be expressed as follows:

(3) $$s_{t,i}\left(t\right)=A_{0,i}\cos\left[2\pi \left(f_{0}t+\frac{1}{2}\beta t^{2}\right)+\phi_{0}\right],t\in \left[0,T_{m}\right]$$

where *A*_0,*i*_ is the amplitude of the transmitted signal; $\phi_{0}$ is the initial phase; *f*_0_ is the carrier frequency; $\beta =\frac{\Delta F}{T_{m}}$ is the frequency modulation slope; ΔF is the frequency modulation offset; and *T_m_* is the period of the modulation signal.

Assuming that the amplitude of the echo signal is attenuated to *A*_1,*i*_, the echo signal *s_r,i_*(*t*) can be expressed as follows:

(4) $$s_{r,i}\left(t\right)=A_{1,i}\cos\left\{2\pi \left[f_{0}\left(t-\tau \right)+\frac{1}{2}\beta {\left(t-\tau \right)}^{2}\right]+\phi_{0}+\varphi_{0}\right\},t\in \left[0,T_{m}\right]$$

where $\tau =2\left(R_{0}+v_{r}t\right)/c$ is the time delay of the target echo signal relative to the transmitted signal, *R*_0_ is the initial distance from the target to the detector, *v_r_* is the relative velocity of the missile-target, *c* is the speed of light, and $\varphi_{0}$ is the phase shift caused by the reflection of the target.

The echo signal *s_r,i_*(*t*) and the transmitted signal *s_t_*_,*i*_(*t*) are mixed and then are filtered by a low-pass filter to obtain the complex form of the point target beating signal *s_b_*_,*i*_(*t*):

(5) $$s_{b,i}\left(t\right)=\frac{1}{2}A_{0,i}A_{1,i}\exp\left[2\pi j\left(\frac{1}{2}\beta \tau^{2}-f_{0}\tau -\beta \tau t\right)+\varphi_{0}\right],t\in \left[0,T_{m}\right]$$

According to the radar equation [22] and the relationship between the signal power *P_t_* and the amplitude *A*_1,*i*_, the point target echo beating signal of each scattering unit is superimposed to obtain the surface target echo beating signal *s_Eb_*(*t*):

(6) $$s_{Eb}\left(t\right)=\sum_{i=1}^{N_{U}}\sqrt{\frac{P_{t}^{2}G_{i}^{2}\lambda^{2}\sigma_{i}}{{\left(4\pi \right)}^{3}\;R_{i}^{4}\left(t\right)}}\exp\left[2\pi j\left(\frac{1}{2}\beta \tau^{2}-f_{0}\tau -\beta \tau t\right)+\varphi_{0}\right]$$

where *N_U_* is the total number of scattering units in the ground echo area, *P_t_* is the power of the transmitted signal, *G_i_* is the antenna gain of the *i* th scattering unit, *σ_i_* is the backscattering coefficient of the scattering unit, *λ* is the working wavelength, and *R_i_*(*t*) is the distance from the *i* th scattering unit to the detector at the current moment.

### 2.2. Construction of Target Echo Signal Model Containing Jamming

By covering the spectrum or time domain energy of the target signals, the blanket jamming *J_Bb_*(*t*) makes the receiver unable to detect the real signal, which requires fast power suppression rather than modulating the real signal [23]. The deceptive jamming *J_Db_*(*t*) is generated by the digital radio frequency memory (DRFM) jammer, which modulates and forwards the stored signal. It is necessary to generate a false signal that is highly similar to the target signal [24]. In this paper, noise amplitude modulation (NAM) jamming, noise frequency modulation (NFM) jamming, sine-wave amplitude modulation (SAM) jamming, and sine-wave frequency modulation (SFM) jamming are selected as four types of blanket jamming [25], and range false target (RFT) jamming and velocity false target (VFT) are selected as two types of deceptive jamming [26].

(7) $$\left\{\begin{array}{c} J_{Bb}\left(t\right)=J_{Sr}\left(t\right)⊗\sum_{i=1}^{N_{U}}s_{t,i}\left(t\right)\\ J_{Db}\left(t\right)=J_{Dr}\left(t\right)⊗\sum_{i=1}^{N_{U}}s_{t,i}\left(t\right) \end{array}\right.$$

Similarly, assuming that the deceptive jamming is effective, there will be a certain time delay between the RFT and the true objective, and the VFT will have a certain frequency deviation. The equations of various types of jamming are shown in Table 1.

In the above table, *U_j_*_1_ is the carrier level of NAM, *u*_n_(*t*) is the modulation noise of NAM; *U_j_*_2_ is the amplitude of NFM; *K_FM_* is the slope of frequency modulation; *u*(*t*) is the modulation noise of NFM; *U_j3_* and *U_j4_*, respectively, represent the amplitude of SAM and SFM; *A_j_* is the amplitude of the jamming signal, $\tau_{j}=2D_{j}/c$ is the time delay after DRFM modulation; *D_j_* is the distance between the detector and the modulated false target; and *f_j_* is the Doppler frequency shift modulation frequency. In order to ensure the effectiveness of the jamming, the center frequency and phase of the blanket jamming carrier are kept consistent with the target echo signal.

The target echo signal with single jamming (*s*_1_) can be obtained by superimposing the beating signals of different jamming in Table 1 with the beating signal of target echo in Section 2.1. The target echo signal with compound jamming (*s*_2_) can be obtained by combining the blanket jamming with the deceptive jamming and superimposing it with the beating signal of the target echo.

(8) $$s_{1}=s_{Eb}\left(t\right)+J_{Bb}\left(t\right)\mathrm{or}\;s_{1}=s_{Eb}\left(t\right)+J_{Db}\left(t\right)$$

(9) $$s_{2}=s_{Eb}\left(t\right)+J_{Bb}\left(t\right)+J_{Db}\left(t\right)$$

## 3. Establishment of Jamming Recognition Model

### 3.1. Extraction of Jamming Recognition Information in Multi-Period “Time Domain + Time-Frequency Domain”

Time domain, frequency domain, and time-frequency domain are often used to analyze signal components and trends in signal processing. The linear frequency modulation signal is an unsteady signal whose frequency changes with time, and its transient and structure are analyzed, respectively, in the time domain and frequency domain. FFT is a commonly used spectrum analysis algorithm, which can use periodicity and symmetry to reduce repeated calculations and improve the real-time calculation efficiency of transient systems [27,28]. The short-time Fourier transform (STFT) takes into account both the time-domain and frequency-domain localization analysis capabilities and is suitable for non-stationary signal processing [29,30]. The time domain, frequency domain, and time-frequency domain of the target echo signal with jamming are analyzed, respectively.

**Figure 2:**
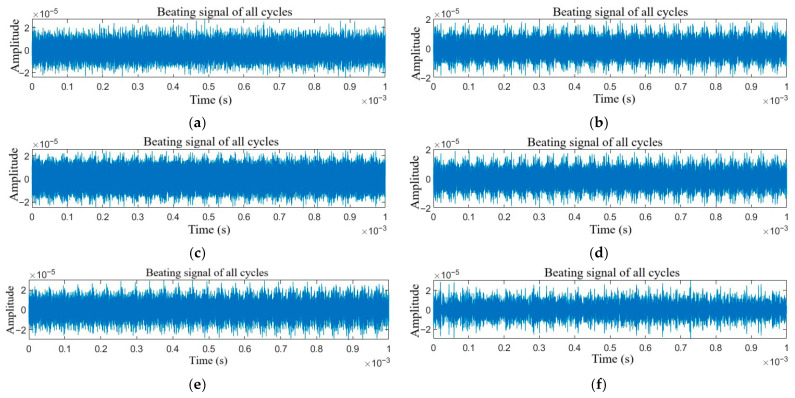
Time domain signals of target echo with single jamming: (**a**) NAM, (**b**) NFM, (**c**) SAM, (**d**) SFM, (**e**) RFT, and (**f**) VFT.

**Figure 3:**
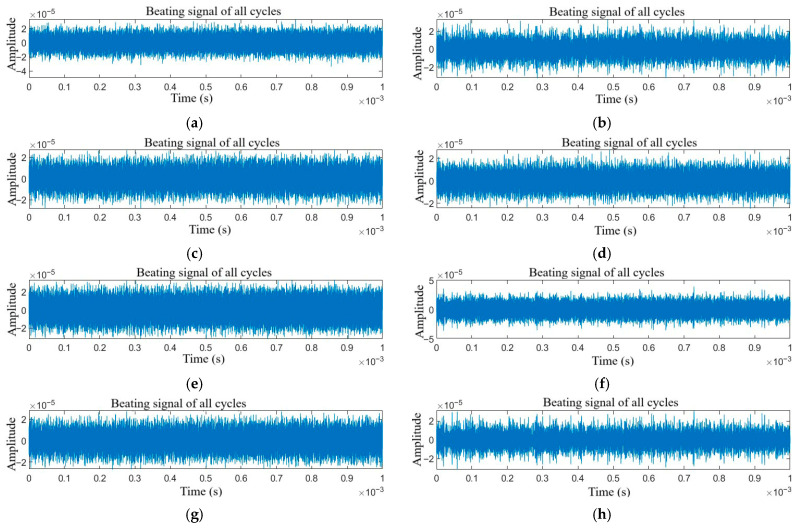
Time domain signals of target echo with compound jamming: (**a**) NAM + RFT, (**b**) NAM + VFT, (**c**) NFM + RFT, (**d**) NFM + VFT, (**e**) SAM + RFT, (**f**) SAM + VFT, (**g**) SFM + RFT, and (**h**) SFM + VFT.

The time-domain signals of target echoes with single jamming and compound jamming are shown in Figure 2 and Figure 3, respectively.

As shown in Figure 2a–f and Figure 3a–h, when different types of jamming are superimposed with the target echo signal, due to the dense frequency characteristics of the multi-period signals and the characteristics of the studied jamming, the differences among various types of jamming are almost indistinguishable.

**Figure 4:**
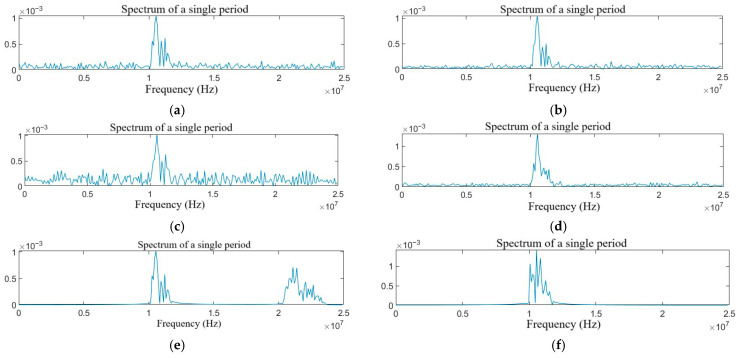
Spectrum diagram of the target echo with single jamming: (**a**) NAM, (**b**) NFM, (**c**) SAM, (**d**) SFM, (**e**) RFT, and (**f**) VFT.

**Figure 5:**
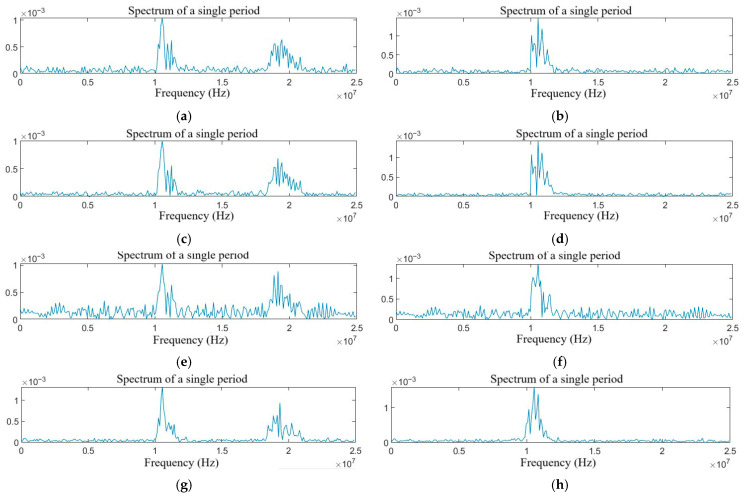
Spectrum diagram of the target echo with compound jamming: (**a**) NAM + RFT, (**b**) NAM + VFT, (**c**) NFM + RFT, (**d**) NFM + VFT, (**e**) SAM + RFT, (**f**) SAM + VFT, (**g**) SFM + RFT, and (**h**) SFM + VFT.

The FFT is used to process the target echo signals with different types of jamming. Based on the symmetry characteristics of the spectrum in a single period, only half of the spectrum is extracted. The spectrum diagrams are shown in Figure 4 and Figure 5.

As shown in Figure 4a–f, the NAM jamming, the NFM jamming, the SAM jamming, and the SFM jamming are all blanket jamming types. When the jamming power is large enough, the target can be completely covered. The RFT jamming has the characteristics of double targets in the frequency domain by changing the echo delay, and other parameters remain unchanged. The VFT jamming is generated by the Doppler frequency shift modulation and forwarding of the intercepted radio signal by the DRFM jammer, which will generate a certain frequency offset in the frequency domain. Because of the limitations of the aircraft speed, the VFT jamming is extremely similar to the echo signal. As shown in Figure 5a–h, the compound jamming manifests as the superposition of the effects of blanket jamming and deceptive jamming.

**Figure 6:**
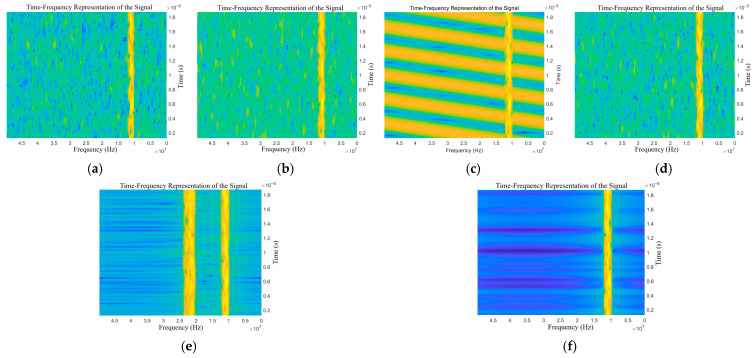
Time-frequency representations of the target echo with single jamming: (**a**) NAM, (**b**) NFM, (**c**) SAM, (**d**) SFM, (**e**) RFT, and (**f**) VFT.

**Figure 7:**
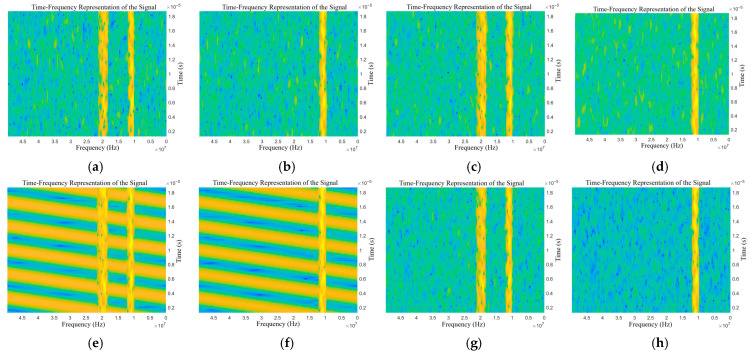
Time-frequency representations of the target echo with compound jamming: (**a**) NAM + RFT, (**b**) NAM + VFT, (**c**) NFM + RFT, (**d**) NFM + VFT, (**e**) SAM + RFT, (**f**) SAM + VFT, (**g**) SFM + RFT, and (**h**) SFM + VFT.

The STFT is used to process the target echo signals with different types of jamming. Similarly, only half periods of the time-frequency spectrum are extracted. The time-frequency representations of the echo signals with different types of jamming are shown in Figure 6 and Figure 7.

As shown in Figure 6a–f, there are obvious differences in time-frequency representation between the two types of deceptive jamming, as well as between deceptive jamming and blanket jamming. There is only a very insignificant texture difference among NAM, NFM, and SFM. As shown in Figure 7a–h, due to the superposition of blanket jamming and deceptive jamming, the characteristics of blanket jamming cannot be distinguished, and there are obvious characteristic differences between deceptive jamming.

**Figure 8:**
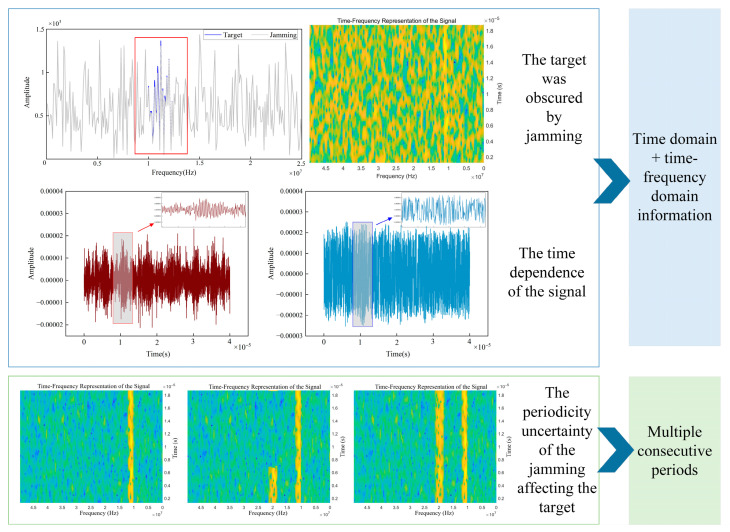
Determination of jamming recognition information.

Since the time-frequency spectrum is a three-dimensional tensor with time-frequency-amplitude coordinates, it has both spectral features and time-varying texture features, thus being regarded as one of the recognition information. However, its texture features are not obvious, especially for compound jamming. Meanwhile, as shown in Figure 8, when the jamming is strong enough to mask the target features, the time-frequency spectrum will lose its distinct recognition features. According to the principles of FFT and STFT, the frequency domain and time-frequency spectrum features cannot be complementary to each other. In order to make up for the lack and deficiency of the time variation pattern, the time domain information is introduced to complement the time-frequency information. The uncertainty of the influence of deception jamming on the echo signal leads to the possibility that the echo signal of a single period lacks effective jamming characteristics. Therefore, multiple continuous periods need to be taken.

In this paper, the time dependence of the time domain waveform is fused with the three-dimensional features of the time-frequency spectrum, and the multi-period “time domain + time-frequency domain” is determined as the recognition information.

### 3.2. Establishment of TFWF-AM Jamming Recognition Models

**Figure 9:**
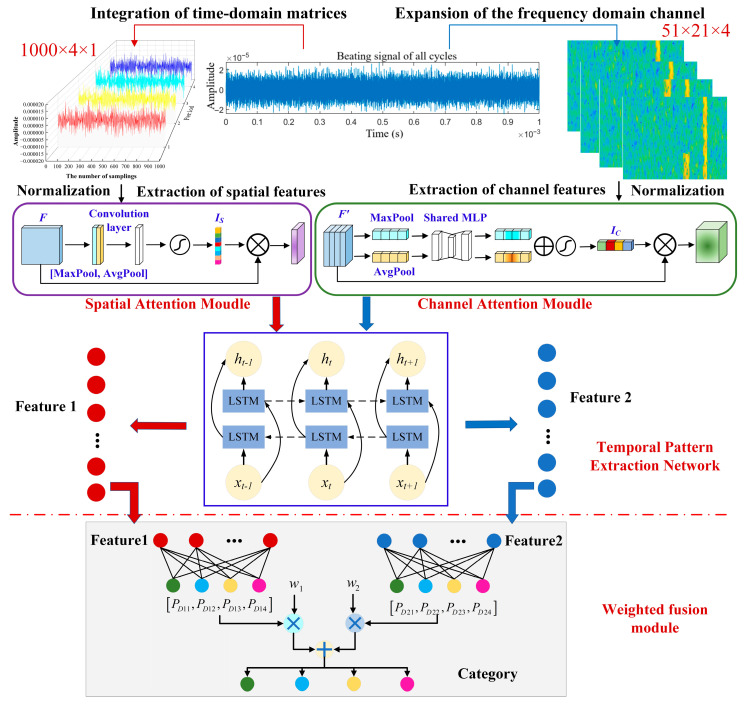
Schematic diagram of the TFWF-AM jamming recognition model.

This model is established based on the CBAM attention mechanism [31,32,33] and the LSTM algorithm [34,35,36]. The spatial attention mechanism is used to extract the features of the time-domain signal matrix at different times, and the channel attention mechanism is employed to extract the features of different channels in the time-frequency spectrum. The bidirectional LSTM is adopted to enable the model to better understand the dependency and patterns of the time series. According to the time domain and time-frequency characteristics of the jamming signal, as well as the principles of different modules of the deep learning network, the jamming recognition model of the time-frequency domain weighted fusion attention mechanism (TFWF-AM) is designed, as shown in Figure 9.

Because of the periodic uncertainty of the jamming affecting the echo signal, a half-period signal of four consecutive periods is processed in this study. First, the four half-period time-domain signals are integrated into a 1000 × 4 time-domain matrix, STFT processing is performed on each half-period signal, and the frequency-domain tensors with four channels are formed by superposition along the channel dimension.

Then, the spatial attention mechanism is utilized to extract the spatial features of the time-domain matrix, and the channel attention mechanism is employed to extract the channel features of the frequency-domain tensor. The principle of the spatial attention module and the channel attention module are Equations (10) and (11), respectively.

(10) $$M_{s}\left(F\right)=Sigmoid\left(f^{7\times 7}\left(\left[AvgPool\left(F\right),MaxPool\left(F\right)\right]\right)\right)$$

where *f* ^7 × 7^ represents a 7 × 7 convolution operation, and [*AvgPool*(*F*); *MaxPool*(*F*)] indicates the concatenation of the results of average pooling and maximum pooling along the channel axis.

(11) $$M_{c}\left(F\right)=Sigmoid\left(MLP\left(AvgPool\left(F\right)\right)+MLP\left(MaxPool\left(F\right)\right)\right)$$

where *F* represents the input feature map, *AvgPool* and *MaxPool,* respectively, denote global average pooling and maximum pooling, and *MLP* is Multilayer Perceptron.

The time-domain spatial features and frequency-domain channel features are input into the bidirectional LSTM, respectively, and the forward hidden layer and the reverse hidden layer are combined to obtain the hidden layer state of the bidirectional LSTM, and the time steps of each channel are weighted and added to generate the fully connected layers, Feature 1 and Feature 2.

(12) $$\vec{h_{t}}=LSTM\left(h_{t-1},x_{t}\right)$$

(13) $$\overset{\leftarrow }{h_{t}}=LSTM\left(h_{t+1},x_{t}\right)$$

(14) $$h_{t}=\alpha \vec{h_{t}}+\beta \overset{\leftarrow }{h_{t}}$$

where $x_{t}$, $\vec{h_{t}}$, and $\overset{\leftarrow }{h_{t}}$ represent the input data, the output of the hidden layer of the forward LSTM, and the reverse LSTM at time *t*, respectively; α and β are constant coefficients, which represent the hidden layer output weight of the bidirectional LSTM, respectively.

The weighted fusion model performs linear transformation on Feature 1 and Feature 2, respectively, and obtains the probability result that the sample is predicted as various types of jamming. The D-S decision is used to weight the two results to determine the final model prediction result. Subsequently, it is determined whether the sample is correctly classified. The weights *w*_1_ and *w*_2_ are adjusted based on the identifiability of individual time-domain information and time-frequency domain information, and the calculation method is as shown in Equation (17).

(15) $$\begin{array}{l} P_{1}=w_{1}\left[P_{D11},P_{D12},P_{D13},P_{D14}\right]+w_{2}\left[P_{D21},P_{D22},P_{D23},P_{D24}\right]\\ =w_{1}\arg\max\left(\mathrm{Feature}1\right)+w_{2}\arg\max\left(\mathrm{Feature}2\right) \end{array}$$

(16) $$C_{1}=\mathrm{MATCH}\left(\max\left(P_{1}\right),P_{1},0\right)$$

(17) $$\left\{\begin{array}{c} w_{1}=\frac{A_{1}}{A_{1}+A_{2}}\\ w_{2}=\frac{A_{2}}{A_{1}+A_{2}} \end{array}\right.$$

where *A*_1_ and *A*_2_, respectively, represent the training accuracy of the model based on single time domain information and single time-frequency domain information.

In this model, the CBAM module enhances the representation ability of key jamming features by dynamically adjusting feature weights through the channel attention mechanism and spatial attention mechanism. The channel attention mechanism generates channel weights through global pooling, while the spatial attention learns the importance of spatial positions through convolution. The two modules work together to enhance the efficiency of extracting local and global features of jamming signals. The bidirectional LSTM layer is used to capture the temporal dependency of jamming signals. The bidirectional structure can simultaneously utilize past and future context information and is applied to the temporal pattern recognition of periodic or sudden jamming (RFT and SFT). Weight fusion is used to dynamically adjust the weights of the two types of recognition information to enhance the robustness of the model.

The channel attention of CBAM can suppress redundant frequency points to highlight the differences in the frequency domain features, enhance the channel characteristics of single-frequency signals, and thereby distinguish between blanket jamming and deceptive jamming. The spatial attention mechanism locates the nonlinearly modulated frequency change area of the instantaneous frequency, which can identify the burst characteristics of frequency hopping, thereby determining the temporal change pattern within the blanket jamming. Further, the bidirectional LSTM is employed for modeling and tracking the temporal continuity of its modulation patterns, capturing its dynamic frequency offset rules, and achieving temporal decoding of the compound jamming.

**Figure 10:**
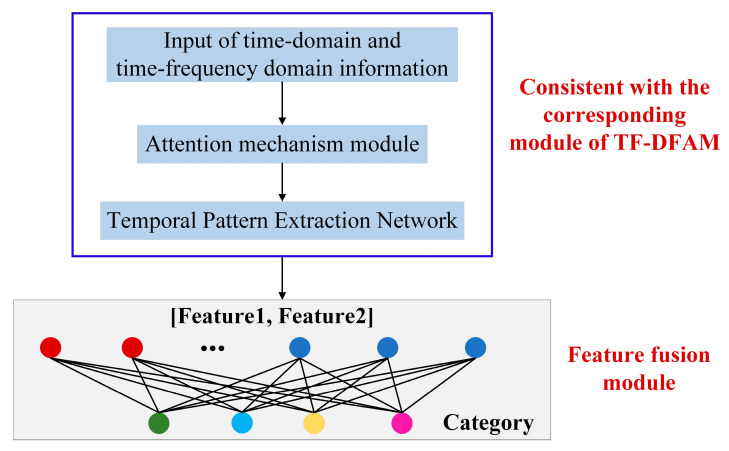
Schematic diagram of the TFFF-AM jamming recognition model.

In order to verify the performance of the model in this paper, a time-frequency domain feature fusion attention mechanism (TFFF-AM) model was established, as shown in Figure 10. The feature fusion model directly concatenates Feature 1 and Feature 2 to form a new fully connected layer. Through linear transformation, the probability that the sample is predicted to be various types of jamming is output. By comparing whether the predicted category index position is consistent with the index position of the label “1”, it is determined whether the sample is correctly classified.

(18) $$P_{2}=\left[P_{F11},P_{F12},P_{F13},P_{F14},P_{F21},P_{F22},P_{F23},P_{F24}\right]$$

(19) $$C_{2}=\mathrm{MATCH}\left(\max\left(P_{F11}:P_{F24}\right),P_{F11}:P_{F24},0\right)$$

The pseudocodes of the two jamming recognition models of TFWF-AM and TFFF-AM are shown in Algorithm 1 and Algorithm 2, respectively.
**Algorithm 1:** TFWF-AM jamming recognition model
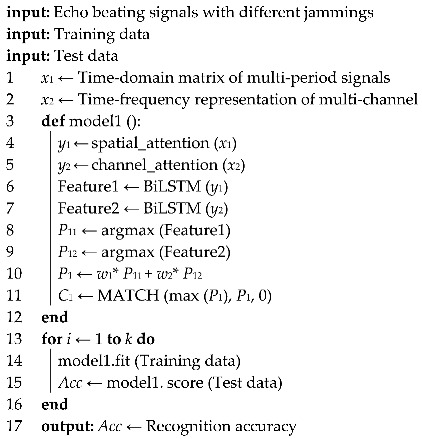


**Algorithm 2:** TFFF-AM jamming recognition model

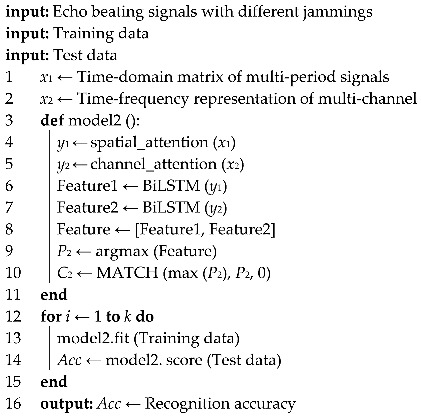



## 4. Establishment of Sample Sets for Jamming Recognition

In Section 2.1, the multi-terrain detection model is established, and then the echo beating signal expression of the surface target is derived. In Section 2.2, the jamming beating signal model is established. Based on the above models, a theoretical echo model will be established using MATLAB R2018b in this section, and the recognition sample sets of different jamming will be generated by superimposing different jamming beating signals into the target echo beating signals. In the process of sample generation, the main parameters of the echo model include carrier frequency, modulation period, etc. The values of the main parameters are shown in Table 2.

The fitting parameters for the backscattering coefficients vary for different terrains. In this study, five terrains of grassland, dry snow, soil and rock surface, short vegetation, and wet snow were adopted. According to the main parameters of the echo model, the fitting parameter values adopted in this paper for the X-band HH polarization mode are shown in Table 3.

Based on the above echo model and parameters, six kinds of single jamming recognition sample sets are generated. The blanket jamming and the deceptive jamming are cross-additively combined to form eight kinds of compound jamming sample sets. The number of recognition sample sets for each type of jamming has been summarized in Table 4.

As shown in Table 4, six kinds of single jamming, namely, NAM, NFM, SAM, SFM, RFT, and VFT, are generated, with 200 samples for each kind of jamming, resulting in a single jamming recognition sample set containing 1200 samples. Eight kinds of compound jamming, NAM + RFT, NAM + VFT, NFM + RFT, NFM + VFT, SAM + RFT, SAM + VFT, SFM + RFT, and SFM + VFT, are generated, and a compound jamming recognition sample set containing 1600 samples is also formed. The sampling time for each sample is 0.001 s. According to the modulation period and the sampling frequency of the beating signal, there are a total of 25 periods, and each period has 2000 sampling points.

## 5. Model Performance Verification

This section uses K-fold cross-validation to verify the performance of the jamming recognition model. For each fold of cross-validation, 200 sets of samples are used as the test set, and all the remaining samples are used as the training set. There is no intersection among the test sets in the K-fold cross-validation. The performance of TFWF-AM and TFFF-AM jamming recognition models is compared, and the accuracy of “time domain + time-frequency domain” jamming recognition information is analyzed, and the model is compared with the existing algorithm model.

### 5.1. Analysis of Recognition Ability of TFWF-AM Jamming Recognition Model

**Figure 11:**
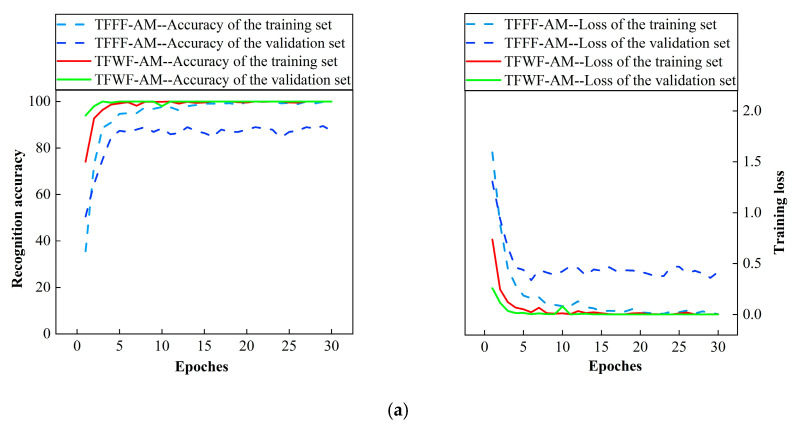

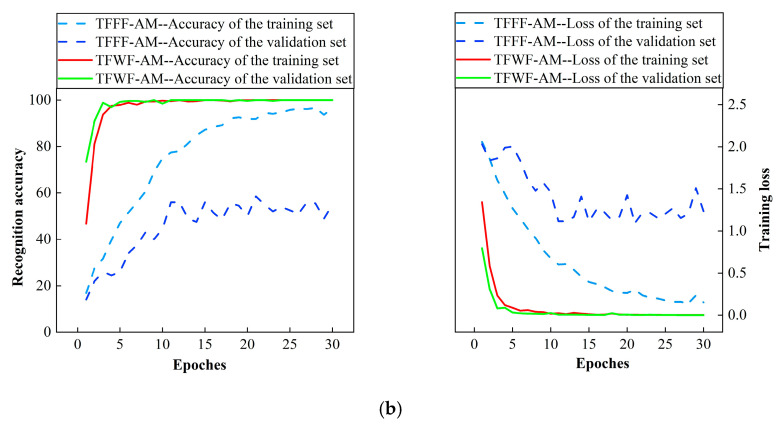
Accuracy graph of training process: (**a**) the recognition accuracy and loss rate of the two models for single jamming and (**b**) the recognition accuracy and loss rate of the two models for compound jamming.

Two models, TFWF-AM and TFFF-AM, are used to identify the single jamming sample set and the compound jamming sample set, and three indexes—the number of misclassifications, the average processing time per batch, and the recognition accuracy of the two models—are compared. The two models are used to identify the single jamming sample set and the compound jamming sample set, and a one-fold cross-validation training process is selected, as shown in Figure 11.

Figure 11 shows the training process of the TFWF-AM and TFFF-AM jamming recognition models for single jamming and compound jamming. During the iterative process, there was a significant disparity between the training set accuracy and the test set accuracy of the TFFF-AM model. Although the training set accuracy was nearly 100% and the loss rate dropped to zero, the test set accuracy was unable to be further improved. While the accuracy and loss curve trend of the training set for the TFWF-AM model is consistent with the test set. Therefore, the TFWF-AM model is more stable than the TFFF-AM model.

**Figure 12:**
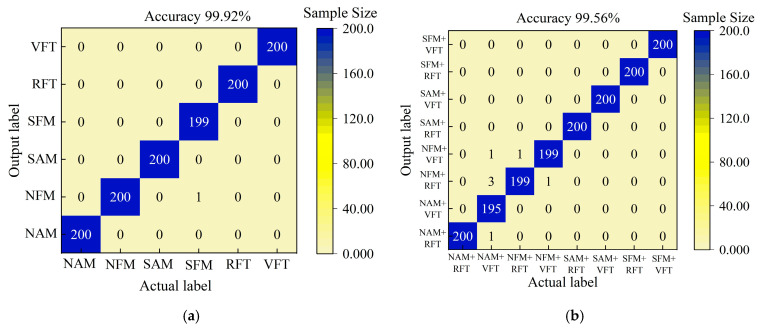

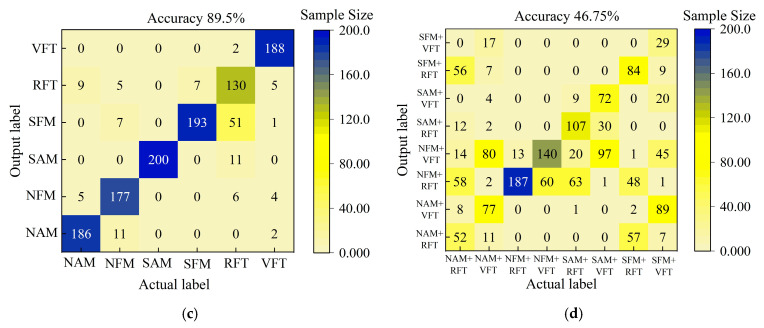
The recognition results of the time-frequency fusion attention mechanism for different jamming: (**a**) the recognition results of the TFWF-AM model for single jamming, (**b**) the recognition results of the TFWF-AM model for compound jamming, (**c**) the recognition results of the TFFF-AM model for single jamming, and (**d**) the recognition results of the TFFF-AM model for compound jamming.

The results of the TFWF-AM and TFFF-AM jamming recognition models for single jamming sample sets and compound jamming sample sets are shown in Figure 12.

The performance of the TFWF-AM and TFFF-AM jamming recognition models was compared in all aspects. The results are shown in Table 5.

Due to the differences in the computing capabilities of the processor hardware, in this paper, the normalized average processing time (NAPT) is used as the real-time performance indicator of the model.

(20) $$\mathrm{NAPT}=\frac{APT}{\mathrm{MAPT}}$$

where APT represents the average processing time per batch of a certain model and MAPT represents the maximum average processing time per batch for the sample set.

As shown in Figure 12 and Table 5, compared with the TFFF-AM jamming recognition model, the TFWF-AM jamming recognition model reduces the number of misclassifications for single jamming and compound jamming by 125 and 845, respectively. Meanwhile, the TFWF-AM model reduced the NAPT per batch by 47.6% and 53.6% for the two sample sets and improved the recognition accuracy by 10.38% and 52.81%, respectively. Therefore, the comprehensive performance of the TFWF-AM jamming recognition model in this paper is superior to that of the TFFF-AM jamming recognition model.

Table 6 analyzes the embeddability of the model.

In terms of computational complexity, the FLOPs of the TFWF-AM compound jamming recognition model proposed in this paper is 100.83 M, which is at the same order of magnitude as the existing lightweight model (MLCJRN) and is significantly superior to other deep learning-based jamming recognition models (MANet and JRNet). In terms of the number of model parameters, the TFWF-AM jamming recognition model has a parameter size of 2.48 M for the compound jamming, which is comparable to that of MANet and much lower than that of JRNet.

Meanwhile, the activation memory requirement of the TFWF-AM algorithm proposed in this paper is approximately 2.94 MB. Taking XC7A100T FPGA as an example, the model (100.83 M FLOPs) mainly consists of convolutional calculations (80%), and each 3 × 3 convolution kernel requires 9 DSP48E1s to perform multiplication operations. According to the design of “processing 8 feature maps in parallel” (balancing delay and resources), only 80–120 DSPs are needed, which is much lower than the 240 DSPs of XC7A100T. The model activation memory (2.94 M) and the parameters (2.48 M) can be fully stored in the on-chip BRAM without relying on external DDR. Therefore, the algorithm proposed in this paper demonstrates strong deployability and applicability in general.

To present the performance of the model more intuitively, this study uses four indicators—Precision, Recall, F1 score, and Accuracy—to evaluate the recognition effect of the TFWF-AM model on various types of jamming and analyze the performance of the model.

As shown in Table 7 and Table 8, when applying the model in this paper to identify single jamming and compound jamming, the four indicators—Precision, Recall, F1 score, and Accuracy—all demonstrated excellent performance.

### 5.2. Accuracy Analysis of Different Jamming Information

This section analyzes the accuracy of multi-period “time domain + time-frequency domain” jamming recognition information. According to the recognition results of Section 5.1, the performance of the TFWF-AM jamming recognition model is better than that of the TFFF-AM jamming recognition model. Moreover, compared to single jamming, the recognition difficulty of the compound jamming sample set is higher. Therefore, this section uses the TFWF-AM jamming recognition model to identify the compound jamming sample set and counts the misclassification quantity of 1600 samples under four recognition information categories: time domain, frequency domain, time-frequency domain, and “time domain + time-frequency domain”.

**Figure 13:**
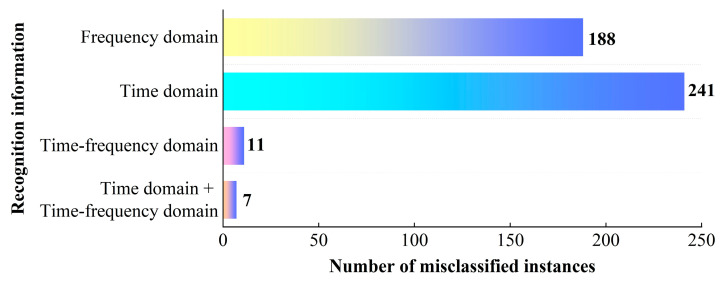
The number of misclassified instances under different jamming recognition information.

As shown in Table 9 and Figure 13, the number of misclassified instances of the compound jamming sample set using time-domain information and time-frequency domain information are 241 and 11, respectively, while the number of misclassified instances using frequency-domain information is 188. Further, the time domain and time-frequency domain information are fused, and the number of misclassified instances of the compound jamming sample set is reduced to 7. Therefore, the time domain and the time-frequency domain can complement each other in terms of jamming information, which proves the effectiveness of the recognition information.

### 5.3. Analysis of the Model’s Robustness

At present, there are intelligent and dexterous jammers, which use DRFM to sample and forward radar signals quickly so as to achieve the dual effects of deception and suppression. In this paper, three kinds of intelligent jamming types in this field are selected, including chopping and interleaving (CI) jamming, intermittent sampling repeater (ISR) jamming, and smeared spectrum (SMSP) jamming, and the robustness of the model is studied. Similarly, 200 samples are generated for each type of jamming, and K-fold cross-validation is performed. The recognition results are shown in Table 10.

The results show that the recognition accuracy of the model for three kinds of jamming reaches 100%, which verifies the effectiveness of the model for intelligent jamming.

In order to analyze the model’s sensitivity to noise, this section analyzes the recognition accuracy of the model for eight compound jamming sample sets under different signal-to-noise ratio (SNR) conditions. The recognition accuracy is shown in Table 11.

As shown in Table 11, in the process of increasing SNR, the recognition accuracy of the model for eight kinds of jamming shows an overall upward trend. When SNR is greater than 0, the recognition accuracy of the model for eight kinds of jamming reaches more than 80%. When the SNR is less than 0, the recognition accuracy is reduced to less than 70%, so it needs to be used in combination with the noise reduction algorithm.

### 5.4. Comparison of This Model with Existing Algorithm Models

In order to further verify the performance of the model, this paper randomly selects two deep learning algorithms, ResNet [40] and AlexNet [41], and selects four machine learning algorithms [42,43,44,45], XGBoost, logistic regression, decision tree, and AdaBoost, to identify the compound jamming sample set. The results are shown in Table 12.

**Figure 14:**
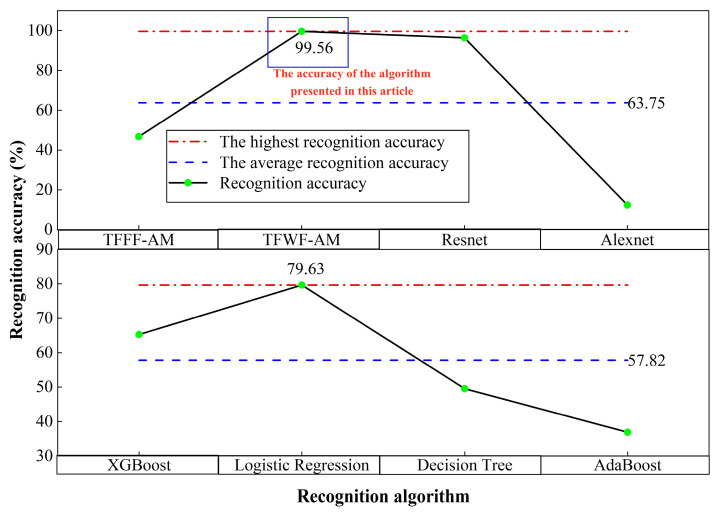
Comparison of this model with existing algorithm models for the compound jamming sample set.

According to Table 12, the recognition accuracy of the compound jamming sample set under four deep learning and four machine learning algorithms is shown in Figure 14.

As shown in Figure 14, the average recognition accuracy of the four deep learning algorithms reaches 63.75%, while the average recognition accuracy of the four machine learning algorithms is only 57.82%. The recognition accuracy of the four deep learning algorithms is compared. The TFWF-AM model proposed in this paper achieved a recognition accuracy of 99.56%, which is much higher than the other three algorithms (ranging from 3.26% to 87.16%).

## 6. Conclusions

Aiming at the problem of insufficient jamming recognition ability of the target detection model, this paper establishes a multi-terrain random fluctuation model, derives the target echo beating signal and multi-jamming beating signal, and carries out the research on jamming recognition based on time-frequency domain fusion and attention mechanism. This paper mainly draws the following conclusions.

1. By analyzing the time domain, frequency domain, and time-frequency domain of the echo signals, the jamming recognition information of “time domain + time-frequency domain” was determined. Combined with the attention mechanism, the TFWF-AM jamming recognition model was established.
2. The TFWF-AM jamming recognition model and the common feature fusion model were compared and analyzed. The TFWF-AM jamming recognition model demonstrated superior performance in terms of the number of misclassified instances, average processing time per batch, and recognition accuracy, and its recognition accuracy for single jamming and compound jamming reached 99.92% and 99.56%, respectively. And this model has excellent embedding capabilities.
3. The number of misclassified instances of the compound jamming sample set by using time-frequency domain information, time domain information, and frequency domain information is 11, 241, and 188, respectively. By constructing the jamming recognition information of “time domain + time-frequency domain”, the number of misclassified instances of 1600 compound samples is reduced to 7.
4. Compared with other jamming recognition models based on deep learning and machine learning, the recognition accuracy of the TFWF-AM jamming recognition model proposed in this paper has increased by 3.26% to 87.16%.

This paper provides a research foundation for the intelligent radio detection system based on real-time jamming sensing and dynamic spectrum modulation. The research on online adaptation, new jamming types, and deployment in real-time radar systems will be conducted in the future.

## Figures and Tables

**Table 1:** Expressions of different types of jamming.

Expressions		
Blanket jamming	NAM	$J_{Sr1}\left(t\right)=\left[U_{j1}+u_{n}\left(t\right)\right]\cos\left(2\pi f_{0}t+\varphi_{Sr1}\right)$
	NFM	$J_{Sr2}\left(t\right)=U_{j2}\cos\left(2\pi f_{0}t+2\pi K_{FM}\int_{0}^{t}u\left(t\right)dt+\varphi_{Sr2}\right)$
	SAM	$J_{Sr3}\left(t\right)=U_{j3}\left[1+m_{j}\cos\left(2\pi f_{j}t\right)\right]\cos\left(2\pi f_{0}t+\varphi_{Sr3}\right)$
	SFM	$J_{Sr4}\left(t\right)=U_{j4}\cos\left(2\pi f_{0}t+m_{f}\sin\omega_{m}t+\varphi_{Sr4}\right)$
Deceptive jamming	RFT	$J_{Dr1}\left(t\right)=A_{j}\cdot \cos\left\{2\pi \left[f_{0}\left(t-\tau_{j}\right)+\frac{1}{2}\beta {\left(t-\tau_{j}\right)}^{2}\right]+\varphi_{Dr1}\right\}$
	VFT	$J_{Dr2}\left(t\right)=A_{j}\cdot \cos\left\{2\pi \left[\left(f_{0}+f_{j}\right)\left(t-\tau \right)+\frac{1}{2}\beta {\left(t-\tau \right)}^{2}\right]+\varphi_{Dr2}\right\}$

**Table 2:** Main parameters of target echo model.

Parameters	Definition	Values
*f*_0_	Carrier frequency	8 GHz
*T*_m_	Modulation period	4 × 10^−5^ s
*v*	Velocity	500 m/s
*c*	Light velocity	3 × 10^8^ m/s
Δ*F*	Frequency modulation deviation	300 MHz
*R*_0_	The initial distance from target to detector	100 m
*P*_t_	Transmission power	1 W
*F_s_*	The sampling frequency of beating signal	50 MHz

**Table 3:** Fitting parameters of the multi-terrain Ulaby model.

Terrains	Incidence Angle (*θ*)		Fitting Value					
			P_1_	P_2_	P_3_	P_4_	P_5_	P_6_
Grassland	0	80	−33.288	32.980	0.510	−1.343	4.874	−3.142
Dry snow	0	75	−13.298	20.048	10.0	4.529	2.927	−1.173
Soil and rock surface	0	80	4.337	6.666	−0.107	−29.709	0.863	−1.365
Short vegetation	0	80	−99.0	97.417	0.114	−0.837	5.0	−2.984
Wet snow	0	80	10.020	7.909	15.0	30.0	0.828	2.073

**Table 4:** Jamming recognition sample sets and quantity statistics.

Type of Jamming		The Quantity of Samples
Single jamming	NAM	200
	NFM	200
	SAM	200
	SFM	200
	RFT	200
	VFT	200
Compound jamming	NAM + RFT	200
	NAM + VFT	200
	NFM + RFT	200
	NFM + VFT	200
	SAM + RFT	200
	SAM + VFT	200
	SFM + RFT	200
	SFM + VFT	200

**Table 5:** Performance comparison of TFWF-AM and TFFF-AM jamming recognition models.

Types of Jamming	Single Jamming			Compound Jamming		
Recognition model	TFFF-AM	TFWF-AM	Parameter comparison	TFFF-AM	TFWF-AM	Parameter comparison
Number of misclassified instances	126	1	−125	852	7	−845
NAPT	1	0.524	−47.6%	1	0.464	−53.6%
Recognition accuracy	89.50%	99.92%	+10.42%	46.75%	99.56%	+52.81%

**Table 6:** Analysis of model embeddability.

Parameters	TFWF-AM	MLCJRN [37]	MANet [38]	JRNet [39]
FLOPs (M)	100.83	108.5	190.0	1820.0
Parameters (M)	2.48	0.98	2.54	11.69
Memory requirements (M)	2.94	-	-	-

**Table 7:** The recognition effect of TFWF-AM for single jamming.

Types of Jamming	NAM	NFM	SAM	SFM	RFT	VFT
Precision	100%	99.5%	100%	100%	100%	100%
Recall	100%	100%	100%	99.5%	100%	100%
F1 score	1.0	0.997	1.0	0.997	1.0	1.0
Accuracy	99.92%					

**Table 8:** The recognition effect of TFWF-AM for compound jamming.

Types of Jamming	NAM + RFT	NAM + VFT	NFM + RFT	NFM + VFT	SAM + RFT	SAM + VFT	SFM + RFT	SFM + VFT
Precision	99.5%	100%	98.03%	99%	100%	100%	100%	100%
Recall	100%	97.5%	99.5%	99.5%	100%	100%	100%	100%
F1 score	0.997	0.987	0.988	0.992	1.0	1.0	1.0	1.0
Accuracy	99.56%							

**Table 9:** The misclassification quantity of different jamming recognition information.

Recognition Information	Time Domain + Time-Frequency Domain	Time-Frequency Domain	Time Domain	Frequency Domain
Misclassification quantity	7	11	241	188

**Table 10:** The recognition results of the TFWF-AM model for the new type of jamming.

Types of Jamming	CI	ISR	SMSP
Sample size	200	200	200
The number of correctly classified samples	200	200	200
Recognition accuracy	100%		

**Table 11:** The recognition results of the model for compound jamming under different SNR.

SNR	−5	−3	0	3	5
Recognition accuracy	46.8%	69.4%	82.4%	80.2%	84.6%

**Table 12:** The comparison of recognition accuracy between this model and the existing algorithm.

Deep Learning Algorithm	Recognition Accuracy	Machine Learning Algorithm	Recognition Accuracy
TFFF-AM	46.75%	XGBoost	65.25%
TFWF-AM	99.56%	Logistic regression	79.63%
ResNet	96.3%	Decision tree	49.5%
AlexNet	12.4%	AdaBoost	36.88%

## Data Availability

The data presented in this study are available on request from the corresponding author. The data are not publicly available due to confidentiality and security restrictions.
